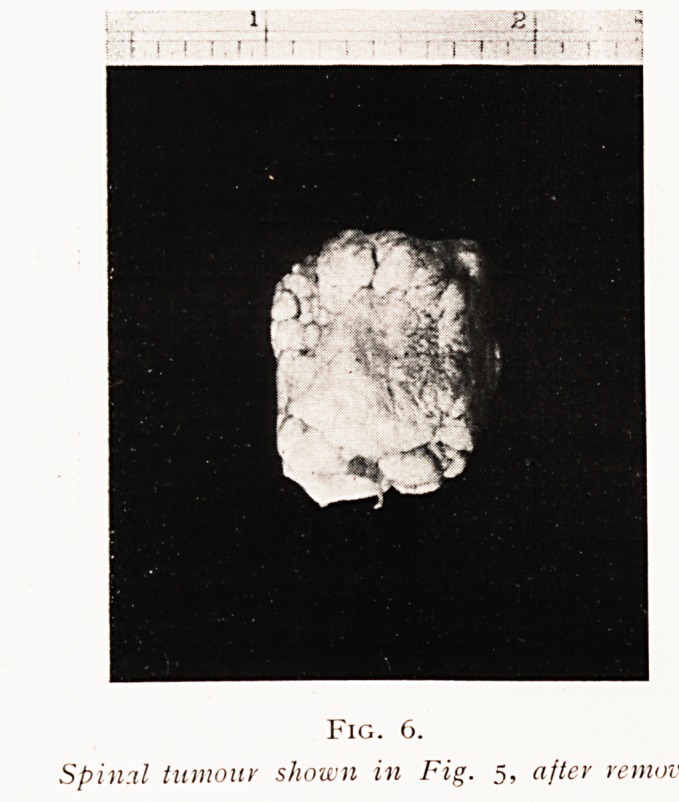# The Present Position of Surgery of the Nervous System
1A Paper read at a Meeting of the Bristol Medico-Chirurgical Society on Wednesday, February 11th, 1925.


**Published:** 1925

**Authors:** Percy Sargent


### THE PRESENT POSITION OF SURGERY OF THE NERVOUS SYSTEM.[1]

BY

Percy Sargent, C.M.G., D.S.O., F.R.C.S.

The developments of neurological surgery during recent years have been so rapid and so important that every practitioner ought to make himself acquainted with them, in order that he may form his own opinion as to their value and be able to offer to his patients such advantages as are to be derived from them.

Successful intervention in disease of the nervous system depends upon accurate diagnosis almost more than in any other region of the body. One may embark upon an abdominal operation with the intention of performing a gastroenterostomy, and end by removing a gall-stone; the failure to make a correct diagnosis beforehand, although regrettable, has done no harm. But with an intracranial

[1] A Paper read at a Meeting of the Bristol Medico-Chirurgical Society on Wednesday, February 11th, 1925.

operation the conditions are entirely different; an exploration of the frontal region when the tumour lies in the cerebellar fossa would not only fail to relieve the patient, but would inevitably leave him worse off than he was before. Recognising this, it seems to me that the surgeon must always lean heavily upon the support of the neurologist. Harvey Cushing, who occupies so prominent a position in the field of neurological surgery, has envisaged a superman so versed in all the clinical and laboratory knowledge of neurology that he can make his own diagnosis, and so skilled in the practical side of surgery that he can perform his own operations. I cannot think this to be anything but a Utopian dream, although we in this country have had in Victor Horsley one who must have come within measurable distance of such an ideal. But a Victor Horsley is born, not made.

It is manifestly impossible for me to do more than indicate briefly what Surgery has to offer in the treatment of some of the diseases of the central nervous system. I have selected four groups of cases which have so far yielded results which can scarcely be ignored even by the most conservative of physicians, namely pituitary tumours, cerebello-pontine tumours, meningeal endotheliomata, and spinal tumours.

1. *Pituitary Tumours.*

There are few things more striking in the whole range of modern physiology and medicine than the advances which in recent years have been made in the pathology of the ductless glands. For many years the thyroid was the only organ of internal secretion of which we had any real knowledge; now the subject of endocrinology has attained stupendous proportions; it is discussed in the public Press, novelists and playwrights make capital out of it, whilst laymen converse freely of the testicle, politely termed monkey-gland, even at meal-times. Whether the pituitary will, like the thyroid, ever be successfully attacked on account of disordered function alone I cannot say. At present its surgical interest lies solely in the symptoms of pressure which are caused by enlargement of the gland or by tumours in its vicinity. The chief of these symptoms are failure of vision and intolerable headache for which relief can be obtained only by operative means, and as far as our present knowledge goes these are the only symptoms for which operation is justifiable.

The tumours which cause these symptoms fall into two main groups, those which arise within the gland and those which originate in its immediate neighbourhood. The former are almost entirely simple adenomatous tumours of the pars anterior ; they excavate the sella turcica so as to be easily recognisable by X-ray examination ; as they invade the cranial cavity, still encapsuled by a dural envelope, they push upwards the optic chiasma and stretch the optic nerves. Characteristic defects of the visual fields appear, and severe tension headaches occur. Generally speaking, the glandular symptoms in these cases are those of deficient secretion or hypopituitarism, namely hebetude, obesity, hairlessness, sexual impotence, increased sugar tolerance, low blood pressure and subnormal temperature. It is a matter of considerable importance to recognise the fact that such patients bear operations badly, and have a remarkably low resistance to microbic attack, so that the glandular symptoms have to be taken into account when an operation is contemplated.

In the second or suprapituitary group we usually find one of two kinds of tumour, the one a meningeal tumour in the interpeduncular space, and the other a tumour or cyst of complex histological structure, probably arising in the infundibulum. These lie above the chiasma and compress it from above downwards (Fig. 1).

Pituitary tumours may be approached either (1) by a temporal operation with elevation of the frontal and temporal lobes, as was practised by Horsley more than twenty years ago; (2) from below through the sphenoidal sinus on the lines

FIG. 1.
*Suprapituitary cholesterin cyst.*

advocated by Cushing; or (3) by turning down a frontal osteoplastic flap hinged in the temporal fossa, and elevating the frontal lobe, as was originally planned by Frazier (Figs. 2 and 3). The last is the method which I am now using, as giving the cleanest and freest exposure of the lesion, and as being applicable to the suprapituitary as well as to the intrapituitary tumour.

*By courtesy of the Editor of The British Journal of Surgery.*—Vol. XI. No. 43, 1924.

FIG. 2.

*Outlines of osteoplastic flap for frontal approach (modified Frazier operation).*

The best results are obtained with the adenomata. I have operated on 20 cases of this nature, 4 by the nasal route and 16 by the frontal, with 7 deaths altogether, and with very gratifying results in the remainder. This mortality of 35 per cent. is being reduced as practice eliminates operative accidents, and as increasing experience

*By courtesy of the Editor of The British Journal of Surgery.*—Vol. XI., No. 43, 1924.

FIG. 3.

*Exposure of tumour by frontal osteoplastic approach.*
*(a) Tumour, (b) Optic nerve and ophthalmic artery.*

allows of the rejection of advanced and unsuitable cases. The last seven patients have made uninterrupted recoveries, with in most cases very striking improvement of vision. Although the mortality is still high, it may confidently be said that the operation of intracapsular removal of an adenomatous pituitary tumour is definitely established as a right and proper surgical procedure.

The suprapituitary tumours are less satisfactory to deal with. Lying as they do in the interpeduncular space in relation to the floor of the third ventricle, they are somewhat less easily accessible, whilst with those which are cystic and contain cholesterine there is the danger of death from what is apparently some form of toxæmia from liberation of their contents into the cerebro-spinal fluid. Amongst my cases there have been five instances of suprapituitary endothelioma and six of infundibular tumour. Seven of the eleven patients survived the operation, and in five of them a substantial degree of improvement was obtained.

2. *Cerebello-pontine or extra-Cerebellar Tumours.*

The next group to which I wish to draw your attention comprises the tumours which are found in the cerebello-pontine angle, and are termed lateral recess tumours or extra-cerebellar tumours. The majority of these are neuro-fibromata of the auditory nerve. Others are the curious " pearl tumours " or cholesteatomata, the origin of which is still uncertain.

The neuro-fibromata are usually firm, solid, encapsuled tumours, but are sometimes quite soft and even cystic. Occasionally they are bilateral and may be part of a diffuse neuro-fibromatosis. Arising just within the internal auditory meatus, they enlarge that orifice, sometimes to a remarkable extent, and on complete specimens of such tumours there can usually be seen a nipple-like projection which has occupied the meatus. As Cushing has pointed out, the enlargement of the internal auditory meatus can sometimes be demonstrated by means of X-rays. This, however, is not a matter of any great importance, as these tumours give rise to such a definite train of symptoms that a precise diagnosis can readily be reached. For a long while, perhaps for many years, deafness and tinnitus may have been the only symptoms complained of, and sometimes the deafness is only discovered accidentally when it is already complete. By Bárány's tests the functional state of the vestibular nerve can be ascertained with precision, and these tests are positive at an early stage in the course of growth of an auditory nerve tumour. Very often the corneal reflex of the same side is diminished or absent, and this is so frequently the case that the corneal reflex as well as the vestibular functions should be tested in all cases of nerve deafness, as the coexistence of these signs would, even in the absence of all other symptoms, be sufficient reason for suggesting the presence of one of these tumours. Sooner or later the tumour, compressing laterally the pons and medulla, blocks the aqueduct of Sylvius, and ventricular distension results. Then appear the classical symptoms of cerebral tumour, the headache, vomiting and papillœdema. When the stage of general increase of intracranial pressure is reached operation becomes at once more difficult and more dangerous, and the outlook more grave.

It might be supposed that an innocent encapsuled tumour, easily diagnosed at an early stage, and surgically accessible without any great difficulty, could be removed completely and safely. This, however, is in my experience unfortunately not the case. It is true that these tumours can be shelled out with ease, but in doing so the medulla, probably through its blood supply, is so damaged that the common result is death from respiratory failure. I have not removed one of these tumours completely since 1921. Up to that time I had so operated upon nine patients, of whom eight died, whilst the only survivor made a complete and excellent recovery and remains well five years later. There is, however, another way of dealing with these tumours, which gives very satisfactory results, and which I have practised for some years, namely the removal of the growth piecemeal from within its capsule. The tumour having been exposed, the capsule is incised, the mass is gradually broken up with a curette, and the fragments are removed by means of a suction apparatus. I have now done 19 such operations with 3 deaths, a mortality of 16 per cent., which is in striking contrast with the 88 per cent. mortality of the operation by total removal.

A further series of 12 cases have been treated by decompression alone, and although one of the patients lived for eighteen years in complete comfort, and then died of an independent malady, these patients have not, generally speaking, been benefited to anything like the same extent as those treated by partial removal, whilst the mortality of the decompression operation was 40 per cent. as compared with the 15 per cent. of intracapsular or partial removal. This difference may be accounted for partly by the fact that most of the cases for which only decompression was done were amongst my earlier operations, and partly because in many of them the intracranial pressure was so far advanced that the tumour could not be exposed.

In these extra-cerebellar cases, then, we have another definite group of intracranial tumours for which surgery offers an increasingly bright outlook. The results can only be improved by earlier diagnosis, which will enable the tumour to be attacked whilst it is still small, and before the intracranial pressure has been raised, before, in fact, the classical symptoms of intracranial tumour have appeared.

3. *Meningeal Tumours.*

The third group, consisting of the endotheliomata, is histological rather than regional. It is regional only in the sense that the tumours are meningeal, and therefore for the most part superficial and more or less easily accessible. They are solid, encapsulated masses which cup but do not infiltrate the brain; they probably arise in the arachnoid tufts which constitute the Pacchionian bodies, and therefore are most commonly found in the neighbourhood of the longitudinal sinus. So slowly do they grow that they may attain very large proportions before giving rise to any symptoms of increased intracranial pressure. The largest which I have yet seen (6½ ounces) was removed from the brain of a patient who had never had a headache and whose optic discs were normal.

I have now operated upon 41 cases of meningeal endothelioma, in 30 of which complete removal was carried out, whilst in 4 only partial removal was possible. In the remaining 7 the tumour was not found, five times on account of incorrect localisation, and twice because the patients died after a "first stage operation." In order to give you as clearly as possible a general impression of the results obtained from these operations I have classed them as follows:—

1. *Recovered completely,* by which I mean that the patients were restored to their normal lives and occupations without any neurological defect. These number 9, approximately 22 per cent.
2. *Recovered,* but remained neurologically imperfect (various palsies, fits and so on). These number 16, approximately 38 per cent.
3. *Died* within 12 months 7, approximately 17 per cent.
4. Post-operative deaths 9, approximately 21 per cent.

Of those patients still alive and well two were operated upon 16 years ago, three 10 years ago, and the rest between 3 and 6 years ago. The longest survival after decompression alone was 16 months.

As these meningeal tumours can often be diagnosed and localised with reasonable certainty, and before signs of increased intracranial pressure appear, they can be removed through an osteoplastic opening. This is a great advantage, as it avoids the very considerable inconvenience of a large cranial defect. Seven of the above-mentioned patients who recovered were operated upon in that manner and consequently have no cranial defect.

Unfortunately, such tumours form only a small proportion of the whole, for of 200 tumours of the forebrain (frontal, temporal, occipital, and parietal) upon which I have operated, no fewer than 160 (80 per cent.) were of an infiltrating or malignant character, the great majority being gliomata. The cerebellar tumours, of which there are 60 instances in my series, are almost exclusively gliomata or gliomatous cysts. It must not be supposed, however, that all these cases of glioma are hopeless and that nothing can be done for them. On the contrary, the results of decompression and of partial removal compare very favourably with the results of many merely palliative operations elsewhere, as for example colostomy for cancer of the colon. Lately we have been burying radium (50 or 100 milligrams for 24 hours) in gliomata with surprising results. The gliomata appear, so far as our observations at present go, to be peculiarly vulnerable to radium.

In operations for tumour of the brain the most important factor upon which hinges success or failure is the degree of intracranial pressure. In the presence of a high degree of pressure the simplest tumour removal is a formidable and dangerous task. In many cases, of course, it is impossible to make a diagnosis of tumour until papillœdema appears, but that and the other signs of increased intracranial pressure should not be allowed to advance far before operation is undertaken. The classical symptoms of cerebral tumour should be regarded rather as danger signals, or, if far advanced, as signs of impending death. We no longer wait for signs of general peritonitis before operating for appendicitis; neither should we postpone operation for cerebral tumour until headache, vomiting and papillœdema are present. We must look for better results not only to the surgeon from improvements of technique, but also and chiefly to the physician from earlier and more accurate diagnosis, as well as in a greater readiness to procure for his patient the only treatment which offers any hope. We are not looking to him in vain. I have had the privilege of working with many physicians, and I have been greatly impressed with the enormous improvement in accuracy of localisation which has been evident during the past few years.

It may be that we shall be able to derive some additional assistance in the future by radiological methods, and on this point I should like to make a few observations.

Ventriculography has now had a fair trial at the hands of several workers, notably Jefferson of Manchester. The procedure is to inject air into the lateral ventricle of the brain, and to take radiograms with the head in various positions so as to ascertain whether one or other ventricle is obliterated, dilated, or otherwise deformed. It has been claimed that tumours, unlocalisable by other means, can be located, and doubtless in some obscure cases this method does afford assistance. As, however, the percentage of cases in which neurological examination fails to localise a tumour is very small, and as the operation is by no means free from danger, its sphere of usefulness is necessarily limited. It has not anything like the same value for the brain as Sicard's radiographic method has for the spine.

4. *Spinal Tumours.*

The fourth group of cases, namely that of spinal tumours, provides some of the most gratifying of all the results of operation upon the central nervous system.

FIG. 4.
*Typical spinal tumour.*

In looking through, as I did recently, many volumes of the *Transactions* of this Society, I was most interested to find in the very first number (1883) the report of a case of Spinal Tumour by Dr. Long Fox, illustrated by an excellent full-page drawing. It is labelled "sarcoma," but it is doubtless what we to-day know as an endothelioma. The drawing differs in no essential respect from the typical picture which I show here (Fig. 4).

Amongst the large number of cases in which I have operated for compression paraplegia there have been 72 instances of spinal tumour. These are grouped as follows :—

| | | |
|---|---|---|
| Intrathecal extramedullary .. .. | | 35 |
| Benign .. .. .. | 29 | |
| Malignant .. .. | 6 | |
| Intramedullary .. .. .. .. | | 16 |
| Extrathecal .. .. .. .. | | 10 |
| Benign .. .. .. | 5 | |
| Malignant .. .. | 5 | |
| Malignant disease of the bone .. | | 11 |
| | | 72 |

Thus of the whole number 29, or more than 40 per cent., were benign extramedullary growths capable of complete removal, and in everyone there must have been some period in which a diagnosis could have been made and operation carried out whilst the cord was still capable of complete and permanent recovery. Such operations are attended with very little risk.

These tumours are capable of diagnosis at an early stage by neurological signs, by the condition of the cerebro-spinal fluid, and by radiography, and there is no excuse for allowing a patient to become totally paralysed and finally to succumb when operation offers the opportunity of a complete and permanent cure. The radiographic method of diagnosis, originated by Sicard of Paris, depends upon the arrest, at the point of blockage, in the spinal canal, of a substance opaque to the X-rays. Such a substance is lipiodol, a heavy inert oil containing iodine, and it is remarkably opaque to the X-rays, more so, in fact, than bone. When it is desired to ascertain the presence or absence, or the level, of a block in the spinal canal, a cubic centimetre of this lipiodol is injected into the cisterna magna through a sub-occipital puncture, the patient being in a sitting position, and radiograms of the spine are taken. If no

FIG. 5.
*Lipiodol arrested at upper limit of tumour.*

obstruction exists the lipiodol falls rapidly to the bottom of the theca, and appears opposite the second sacral vertebra as a rounded or conical shadow. If, however, a tumour, abscess, or other block is present, then the lipiodol is shown arrested at the upper level of the obstruction (Fig. 5). This method affords a valuable means of diagnosis in cases of spinal tumour, for it not only shows the presence of a block in the spinal cord, but also demonstrates the exact level of the obstruction with relation to the bones, and enables the surgeon to plan his laminectomy with the greatest accuracy (Fig. 6). It is only the upper level, however, which is thus demonstrated. If it is desired to show the lower level of the tumour, or in other words to estimate its longitudinal extent, a thing which neurological examination does not enable us to do, then the lipiodol injection is made by

FIG. 6.
*Spinal tumour shown in Fig. 5, after removal.*

lumbar puncture, and the radiograms taken with the patient in the head down position.

As we become more familiar with the interpretation of lipiodol-radiograms, we shall doubtless be able to derive information regarding other spinal lesions such as chronic meningitis and meningitis circumscripta. It has already proved of great value in traumatic cases when the question of laminectomy has to be decided.

## Figures and Tables

**Fig. 1. f1:**
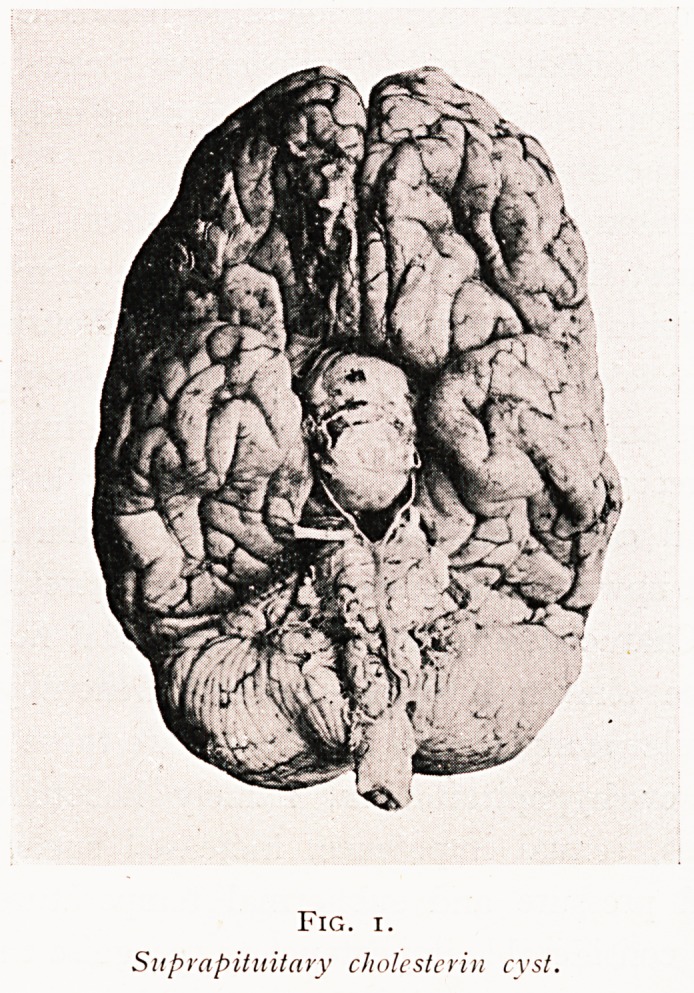


**Fig. 2. f2:**
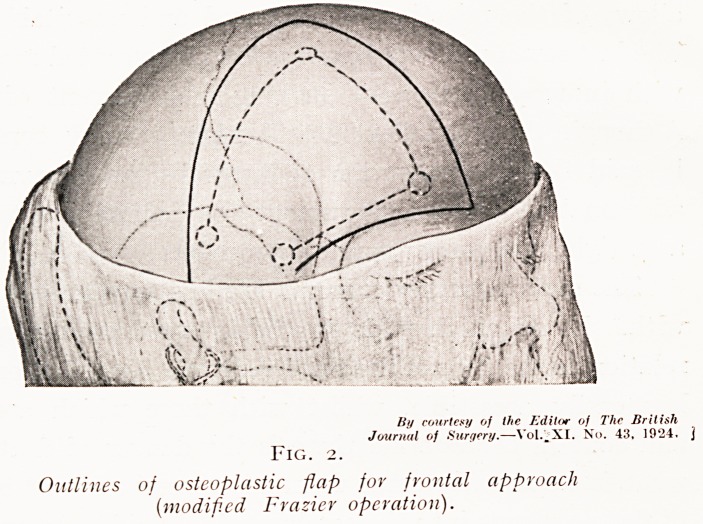


**Fig. 3. f3:**
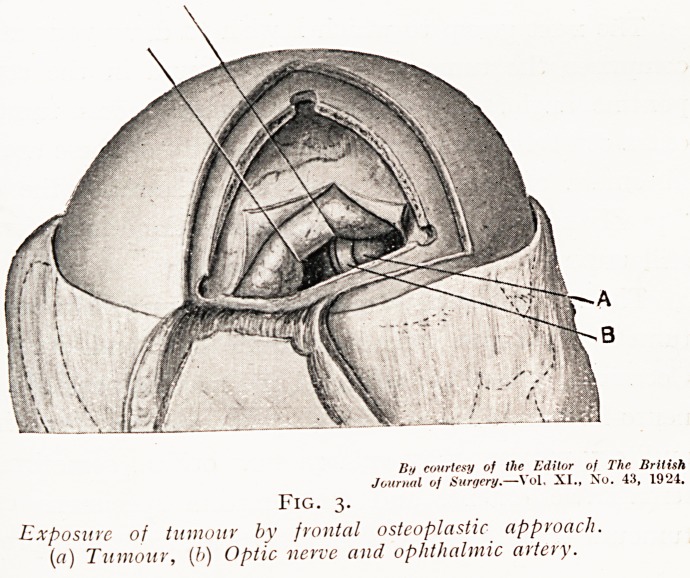


**Fig. 4. f4:**
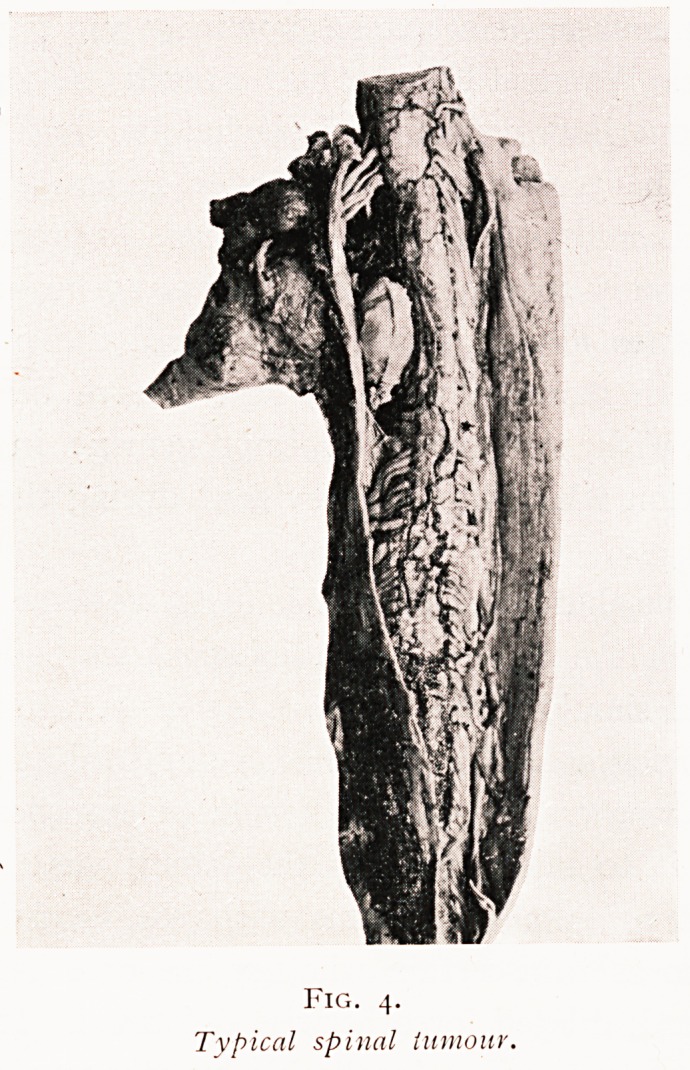


**Fig. 5. f5:**
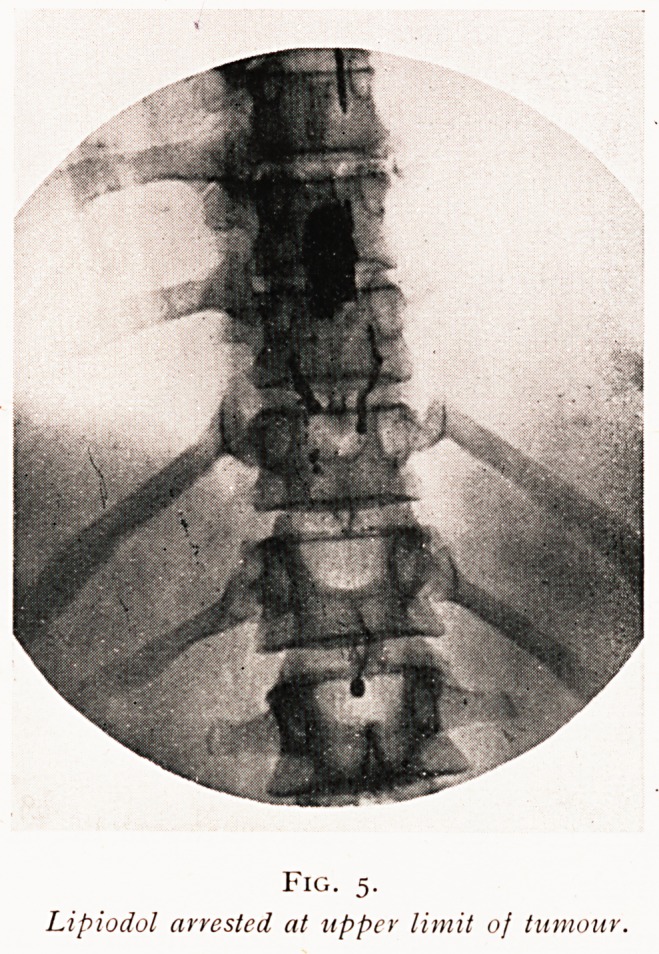


**Fig. 6. f6:**